# Evaluating a Web-Based Mental Health Service for Secondary School Students in Australia: Protocol for a Cluster Randomized Controlled Trial

**DOI:** 10.2196/12892

**Published:** 2019-05-17

**Authors:** Bridianne O'Dea, Catherine King, Mirjana Subotic-Kerry, Melissa Anderson, Melinda Rose Achilles, Belinda Parker, Andrew Mackinnon, Josey Anderson, Nicole Cockayne, Helen Christensen

**Affiliations:** 1 Black Dog Institute Sydney Australia; 2 Faculty of Medicine University of New South Wales Sydney Australia; 3 Centre for Mental Health University of Melbourne Melbourne Australia

**Keywords:** schools, adolescent, mental health, depression, anxiety, mental health services, internet

## Abstract

**Background:**

Mental health problems are prevalent among Australian secondary school youth; however, help-seeking is low. Schools offer an ideal setting to address these concerns. The Black Dog Institute has developed a Web-based mental health service for secondary schools that is modeled on the principles of stepped care. The Smooth Sailing service aims to improve help-seeking and reduce anxiety and depressive symptoms in secondary school students. The acceptability of this service has been demonstrated in a pilot study. A full trial is now warranted.

**Objective:**

This study protocol for a cluster randomized controlled trial (RCT) aims to evaluate the effectiveness of the Smooth Sailing Web-based service for improving help-seeking intentions and behavior, and reducing depressive and anxiety symptoms, alongside other mental health outcomes, when compared with a school-as-usual control condition in secondary school youth.

**Methods:**

This RCT aims to recruit 1600 students from 16 secondary schools in regional and urban locations throughout New South Wales, Australia. Schools are randomly assigned to the intervention or school-as-usual control condition at the school level. Approximately 100 students from 1 or multiple grades are recruited from each participating school. Participants complete measures at 3 timepoints: baseline, 6 weeks post, and 12 weeks post, with the primary outcome assessed at 12 weeks posttest. Participants assigned to the intervention condition register to the Web-based service at baseline and receive care in accordance with the service model. Participants in the control condition receive school-as-usual.

**Results:**

The first baseline assessment occurred on February 22, 2018, with the 12-week endpoint assessments completed on Friday, June 29, 2018. Control schools are currently receiving the service, due for completion by June 30, 2019. The trial results are expected to demonstrate improved help-seeking intentions and behavior among students assigned to the intervention condition, alongside improvements in symptoms of depression, anxiety, distress, and other mental health outcomes when compared with students assigned to the control condition.

**Conclusions:**

To our knowledge, this is the first time that a Web-based mental health service based on the principles of stepped care will have been integrated into, and evaluated in, the Australian school context. The findings of this trial will have implications for the suitability of this type of service model in Australian schools and for the delivery of school-based mental health services more broadly.

**Trial Registration:**

Australian New Zealand Clinical Trials Registry ACTRN12618001539224 https://anzctr.org.au/Trial/Registration/TrialReview.aspx?id=375821&isReview=true (Archived by WebCite at http://www.webcitation.org/77N3MDGS6)

**International Registered Report Identifier (IRRID):**

DERR1-10.2196/12892

## Introduction

### Background

Adolescence is a key developmental period for mental illness with 50% of all mental disorders emerging before the age of 18 years [1]. As most young people spend these years in secondary education, schools now play an increasing role in addressing the mental health needs of their students. Schools are ideal settings for recognizing the early behavioral and emotional signs of mental illness among students and for trusted adults to initiate help-seeking [2]. This is important as youth are reluctant to seek formal care [2,3], despite the negative impacts of poor mental health on social and educational functioning [4-7]. Schools have initiated a range of programs aimed at reducing mental illness and improving mental health literacy among students, as well as increasing their likelihood of seeking help [8-11]. However, many of these programs lack evidence or have not had substantial uptake because of implementation barriers [12]. A total of 2 major challenges remain: identifying the students who are experiencing mental health problems and delivering care to those who require it.

Currently, most schools utilize a wait-to-act model in which the staff refer students to school counselling services, learning support or well-being teams after observing concerning behaviors or from students’ self-disclosures [13]. A more proactive approach, one that incorporates mental health screening and automatic stratification and response based on symptom severity, may improve schools’ capacity to address students’ mental health needs. Web-based stepped care presents a viable option for providing this type of care. This is based on the premise that simple, cost-effective Web-based psychotherapy is offered to youth with mild-moderate symptoms, with more costly, intensive face-to-face interventions reserved for those with more severe and persistent symptoms [14]. Although complex, this approach can be efficient, as it provides tailored help as required and aims to prevent serious mental illness by detecting symptoms early [15,16]. This type of care is also equitable, providing all students with an opportunity to have their mental health needs addressed. Internet screening and Web-based cognitive behavioral therapy (CBT) programs can be readily integrated into such a model as these require minimal human input, are acceptable to youth, preserve fidelity of care, and allow for ongoing monitoring and automated feedback [17]. Notably, this type of care system is engineered to reach out to youth rather than wait for them to approach.

Codesigned with students, school counsellors [18], general practitioners [19], and parents [20], the Black Dog Institute has developed a Web-based mental health service called Smooth Sailing. On the basis of the principles of stepped care, Smooth Sailing uses a website to screen, assess, allocate, and deliver psychological interventions to improve help-seeking for mental health problems and reduce depressive and anxiety symptoms among secondary school youth. This service uses brief, validated self-report measures for anxiety and depression [21,22] to accurately determine young people’s symptom severity and their required level of care. The service has 3 degrees of treatment intensity that are consistent with Australian Clinical Practice Guidelines [23]: self-directed Web-based psychoeducation for students with nil to mild symptoms, self-directed Web-based CBT for students with moderate symptoms [9,10], and a direct link to face-to-face care with a school counsellor for students with moderately severe to severe symptoms. Students are monitored fortnightly using a brief survey delivered via short message service (SMS) text messaging or email. Every 6 weeks, students complete a step assessment from which care is reallocated based on their results. If a student reports having thoughts of self-harm or death during any of the assessments, the school counsellor receives an automatic notification.

**Figure 1:**
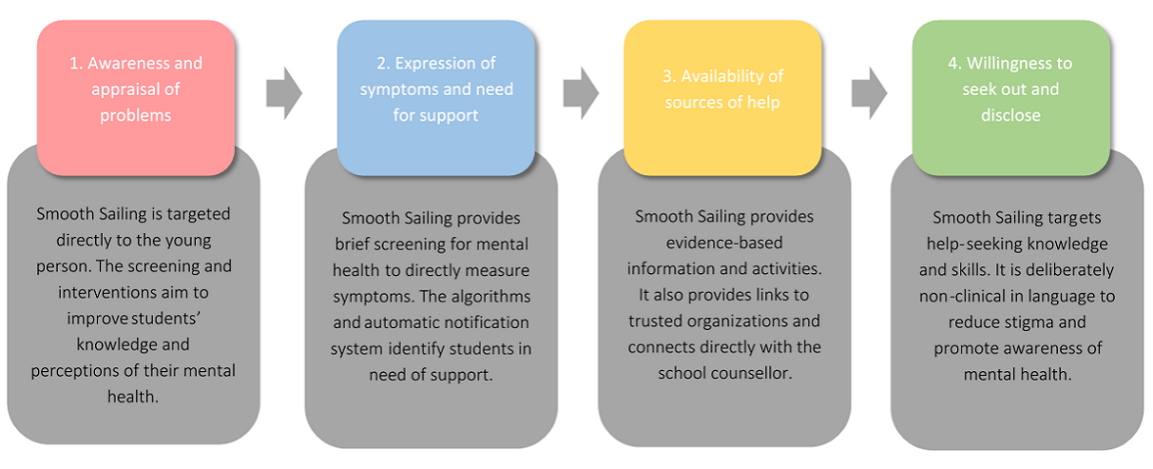
Applying Rickwood et al’s help-seeking model to the Smooth Sailing service.

The Smooth Sailing service is designed as a universal intervention to improve young people’s attitudes toward seeking help for mental health problems. School-based screening has increased referral rates to health care services, demonstrating the positive impact that schools can have on initiating access to care [24,25]. Establishing healthy attitudes to help-seeking for mental health in adolescence, particularly from adults and professionals, is key to supporting lifelong mental health. A number of studies have revealed an association between positive attitudes to professional help-seeking and increased help-seeking intentions and behaviors in adolescents [26,27], university students [28], and adults [29]. The Smooth Sailing service is based on Rickwood et al’s [30] model in which help-seeking is defined as a process with 4 key stages. Outlined in Figure 1, Smooth Sailing attempts to target each of these stages through a range of different functions and content.

A 6-week single-arm pilot study of the Smooth Sailing service was undertaken in 4 New South Wales (NSW) secondary schools in 2017 with 59 students taking part. At posttest, 93% (55/59) of students remained enrolled in the service. The service was found to be acceptable and feasible to students, parents, and school counsellors. Students followed up by the school counsellor reported significant symptom improvements at posttest. Importantly, most of the participants found the service easy to understand (96%, 53/55), easy to use (95%, 52/55), and enjoyable (89%, 49/55). The majority were also comfortable being followed up by the counsellor (82%, 45/55), were likely to use the service again (73%, 40/55) and would tell a friend to use the service (85%, 47/55). However, key questions remain because of the small sample size and lack of a control group. Despite the promise and practicality of the proposed service model, the effectiveness of universal screening, stepped care, and active follow-up from school counsellors for improving young people’s help-seeking and reducing symptoms is still unknown. It is now timely to examine the primary effects of this type of service in comparison with a control group.

### Objectives

The main objective of this trial is to evaluate the effectiveness of the Smooth Sailing service for improving help-seeking intentions (primary outcome), as well as for improving help-seeking behavior, reducing symptoms of depression, anxiety, distress, and barriers to care, and improving mental health literacy/stigma (secondary outcomes) among secondary school students, in comparison with a school-as-usual control group.

### Hypotheses

The primary hypothesis is that those who receive the Smooth Sailing service will report higher scores of overall help-seeking intentions at 12 weeks posttest compared with baseline. The secondary hypothesis is that compared with students in the control condition, those assigned to intervention will report improved help-seeking behavior and reduced levels of depression, anxiety, distress, and barriers to care, as well as improved mental health literacy and stigma at 12 weeks posttest.

## Methods

### Trial Design

This clinical trial protocol adheres to the Standard Protocol Items: Recommendations for Interventional Trials 2013 guidelines [31] (see Table 1). This study is a 2-arm 12-week cluster randomized controlled trial (RCT). Data are collected at 3 intervals: baseline, 6 weeks, and 12 weeks posttest with the primary outcome measure assessed at 12 weeks posttest.

### Randomization

Assignment of schools to the control or intervention condition is carried out according to the International Council for Harmonisation guidelines [32] and performed by a researcher not involved in the day-to-day conduct of the trial. Schools are allocated to a single condition (cluster design) to avoid contamination and for administrative convenience [33]. A minimization approach [34,35] is used to ensure balance across conditions in terms of the Index of Community Socio-Educational Advantage level (<1000 vs ≥1000), gender mix (coeducational vs single sex), and year level involved (year 9 students only vs multiple or other years). Minimization is undertaken in StataCorp LLC Stata statistical software version 14.2 using the rct_minim procedure [36]. The pool of already available schools is sorted in random order using the Excel 2003 data analysis random number generator and entered into the minimization routine in ascending order using the factors specified above. Subsequent schools are assigned to an intervention arm using rct_minim in the order that they join the trial and provide complete information. As this trial uses a no-treatment comparator control group, participants and researchers are not blinded to the allocation assignment.

### Ethics Approval

Ethics approvals are obtained from the University of New South Wales (UNSW) Human Research Ethics Committee (HREC; HC17910), the State Education Research Applications Process (SERAP) for the NSW Department of Education (SERAP 2016471), the Sydney Catholic Schools (SCS) Research Centre (20186), and the Catholic Schools Office Diocese of Maitland-Newcastle.

### Setting

This trial is conducted in government, independent, and Catholic secondary schools located throughout NSW, Australia.

### Participants

#### Inclusion Criteria

All secondary students from the participating year groups, aged between 11 and 19 years, who attend one of the participating schools, are invited to participate. Both males and females are eligible. Participants are required to have an active email address for the duration of the trial. Only those students who can read and understand English and provide their signed written consent are able to participate in the research study and use the service. All participants can access their own mental health support or treatment throughout the trial.

#### Exclusion Criteria

Schools are required to have a school counsellor available onsite for the school visits and for the duration of the study.

**Table 1:** Standard Protocol Items: Recommendations for Interventional Trials Compliance: Items from the World Health Organization dataset.

Data category	Information
Primary registry and trial identifying number	Australian New Zealand Clinical Trials Registry (ANZCTR): ACTRN12618001539224
Date of registration in primary registry	September 14, 2018
Secondary identifying numbers	—^a^
Source(s) of monetary or material support	Hong Kong and Shanghai Banking Corporation
Primary sponsor	Black Dog Institute, University of New South Wales
Secondary sponsor(s)	—
Contact for public queries	Dr Bridianne O’Dea b.odea@blackdog.org.au
Contact for scientific queries	Dr Bridianne O’Dea b.odea@blackdog.org.au
Public title	Smooth Sailing: Evaluating an online service for student well-being
Scientific title	Smooth Sailing: Evaluating an online service for student well-being
Countries of recruitment	Australia
Health condition(s) or problem(s) studied	Depression, anxiety, help-seeking for mental health
Intervention(s)	Intervention: A Web-based school-based mental health service for secondary students (Smooth Sailing); based on the principles of stepped care; 12-week duration
Control	Control: school-as-usual; waitlist
Key inclusion and exclusion criteria	Inclusion: Male and female secondary students; currently attending high school at 1 of the participating schools; have an active email address for the duration of the trial; Exclusion criteria: nil
Study type	Study type: Interventional; Allocation: Randomized; Intervention model: Parallel assignment; Primary purpose: Prevention
Date of first enrolment	February 22, 2018
Target sample size	1600
Recruitment status	Completed
Primary outcome(s)	Help-seeking intentions (measured using the General Help-Seeking Questionnaire). Primary timepoint: 12-week endpoint
Key secondary outcomes	Help-seeking behavior (measured using the Actual Help-Seeking Questionnaire); Symptom levels of: Distress (measured using the Distress Questionnaire-5); Depression (Center for Epidemiologic Studies Depression Scale-Child Version); Anxiety (measured using the Generalized Anxiety Disorder Questionnaire 7-item); Mental health literacy and stigma (measured using the Mental Health Literacy and Stigma Scale); Barriers to seeking help (measured by the Barriers to Seeking Help-Brief Scale); Functioning (measured by 1-tem scale). Primary timepoint: 12-week endpoint
Ethics review	Approved by University of New South Wales Human Research Ethics Committee on November 24, 2017
Completion date	June 29, 2018
Summary results	Data currently under analysis
Individual participant data sharing statement	Plan to share individual participant data (No)

^a^Not applicable.

### Recruitment

**Figure 2:**
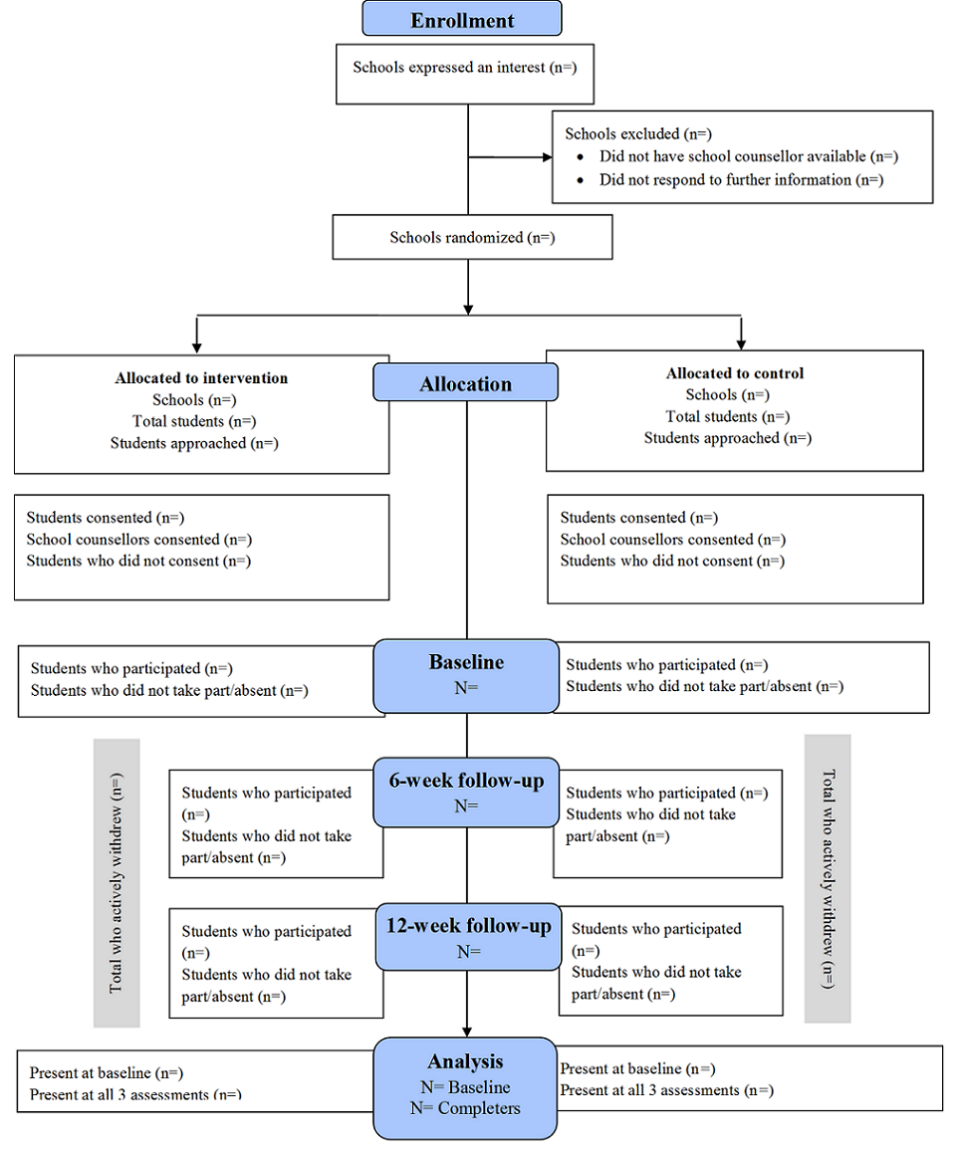
Consolidated Standards of Reporting Trials flow diagram that will be used to illustrate participation throughout the phases of the Smooth Sailing Cluster Randomized Controlled Trial.

The flow chart used to outline recruitment, randomization, and participation for this trial is provided in Figure 2.

To recruit schools, the study advertisement is circulated by email to NSW school counsellors who subscribe to the Black Dog Institute’s mailing list. Counsellors are invited to express an interest in the study by contacting the chief investigator (CI) by email. The CI responds to these emails with a study information pack and schedules a phone call to explain the study process and answer additional questions. Schools are informed that the study is available to students in any year group but is considered by the research team to be well suited to students in Grade 9 (ages 14 to 15 years), as half of all lifetime mental disorders emerge during the mid-teens [1]. To recruit students, a set of Student Information Forms are mailed to each school and distributed to students by a school staff member before the baseline assessment. Interested students are asked to review the material, discuss with their parents/guardians, and consider study participation.

### Consent

For school consent, schools are asked to provide a signed letter of support from the school principal. This letter is then forwarded to the governing ethics bodies to confirm school participation. Individual student consent is provided by a signed copy of the Participant Information and Consent Form (PICF) that is obtained on the day of the baseline assessment. For all schools other than those in the Sydney Catholic Diocese, an opt-out consent process is employed for parental consent. In these schools, parents are notified of the study using the school’s usual methods of communication (eg, school newsletter). Parents are given 14 days to inform the school if they do not wish for their child to participate and can withdraw their child at any time. Students from schools in the Sydney Catholic Diocese are required to obtain signed parental consent. The PICFs for SCS are distributed to students 2 weeks before the baseline assessment. On the day of the baseline visit, PICFs are checked by the researchers to ensure student and parent consent is provided.

### Withdrawal of Consent

The PICF informs participants that taking part is completely voluntary and that they are free to withdraw from the study at any time without penalty and without having to give a reason. Participants can withdraw by emailing the research team or notifying the researchers at the school visits. Parents can also withdraw their child at any time using the same methods or by contacting their child’s school.

### The Intervention

The Smooth Sailing service is accessed using a website that requires students to register using a unique code. At registration, students complete an additional consent measure (a 6-item Gillick Competency Test) to ensure they understand the terms and conditions of the service. After correctly completing this measure, students are asked to input their code, provide their email address, mobile phone number (optional) and create a password. Once registered, the students complete a step assessment. This consists of reliable and valid measures of depression and anxiety symptoms [21]. The 9-item Patient Health Questionnaire-9 (PHQ-9) [21] is used to measure the presence of depressive symptoms over the past 2 weeks, with items rated on a scale from not at all (0) to nearly every day (4). The service also uses the Generalized Anxiety Disorder Scale-7 (GAD-7) [22] to measure the presence of anxiety symptoms in the past 2 weeks using the same response options as the PHQ-9. Students’ total scores on the PHQ-9 and GAD-7 scales are used to allocate them to a step of care (see Table 2).

After the step assessment, the Smooth Sailing service produces a personalized dashboard that provides students with an overview of their recommended activity. The Web-based psychoeducation consists of 5 10-min modules that provide information about anxiety, depression, and help-seeking (see Table 3). The modules were created specifically for the Smooth Sailing service and were reviewed in the co-design process by young people as well as a clinical psychologist. The content was also edited by a copywriter to ensure it was written at an appropriate reading level. The modules are complemented by animations and illustrations as well as hyperlinks to other credible youth mental health services and websites. All modules are self-paced and can be completed in any order. Module 6 includes referral to 2 Web-based, publicly available, free evidence-based CBT programs for depression and anxiety [37-39]. MoodGym [40] comprises 5 modules in which young people learn a range of strategies to identify and manage unhelpful patterns of thinking, connect their thoughts to their feelings, reduce worry, and improve self-esteem and interpersonal relationships. The BRAVE Program [41] includes 10 1-hour self-directed sessions that are usually completed over 10 weeks that teach young people to identify anxiety and stress, develop relaxation and problem-solving skills, and reframe negative thinking. These programs are provided to any student who is allocated to Steps 2, 3, and 4 throughout the study period.

**Table 2:** Smooth Sailing service model: step criteria and intervention provided.

Criteria		Step				
		0	1	2	3	4
Score range		0-4	5-9	10-14	15-19	20+
Symptom severity		Nil	Mild	Moderate	Moderately severe	Severe
**Interventions provided**						
	Web-based psychoeducation	Yes	Yes	Yes	Yes	Yes
	Self-directed Web-based cognitive behavioral therapy	No	No	Yes	Yes	Yes
	Face-to-face sessions with a school counsellor to provide counselling and/or referral to external services	No	No	No	Yes	Yes

**Table 3:** Module overviews.

Title	Content description
What is mental health?	Information about mental health issues that are common among youth and when it might be time to seek help
Feeling on edge	Information on anxiety, how to identify it, potential causes, where to seek help, and practical tips for managing it
Waves of sadness	Information on depression, differences between sadness and depression, potential causes, how and where to seek help, and practical tips to cope
When it’s time to tell someone	Information about when to seek help, how to talk to friends and parents, seek help from a general practitioner, and the roles of different health professionals
When a mate needs a hand	Ways to help others including having a private chat, seeking help together, respecting the treatment process, and the importance of looking after yourself
Don’t fret, help is here	Offers access to 2 evidence-based, free Web-based cognitive behavioral therapy programs, produced by Australian universities. Young people can select which program they prefer

In the Smooth Sailing service, school counsellor follow-up notifications are initiated if a student is allocated to Steps 3 or 4 or if a student reports having thoughts that they would be better off dead or thoughts of hurting themselves in the past 2 weeks (ie, score >0 on item 9 of the PHQ-9). School counsellors conduct the follow-up sessions during school time but after completion of the researcher visit. School counsellors follow their normal school protocols and duty of care procedures when attending to the notified students, initiating external referrals and parental contact when necessary.

For the duration of the study period, the Smooth Sailing service sends a fortnightly check-in survey to participants via email or SMS text messaging. This consists of the PHQ-2 and GAD-2 and is designed to test participants’ engagement and adherence to monitoring. These are ultrabrief versions of the longer counterparts and measure the presence of depression and anxiety symptoms in the past 2 weeks, with responses scored the same as the full scale [42]. Total scores range from 0 to 12. Users are reminded to use the Smooth Sailing program through email and SMS text messaging notifications that are sent fortnightly, on alternate weeks to the check-in survey. Every 6 weeks, a step assessment is completed which reallocates the level of care, depending on participants’ results. In accordance with the Australian Clinical Practice Guidelines for the Treatment of Depression in Adolescents and Young Adults [23], if a participant has not responded (ie, symptoms remain elevated or have worsened) within 6 weeks, they are stepped up to the next level of care. The combined step allocation accounts for the participants’ previous step, their current step, and whether there has been any change (See Table 4 for stepping decisions). Given that the service is in its infancy and its effectiveness is yet to be established, there is no stepping down in the current model. As such, no care is removed from participants, and they are instructed to continue using the service as advised by their updated personalized dashboard. Participants who show improvements can continue to use the Web-based CBT programs and all other modules as they wish.

The data collected by the service are stored securely on the Black Dog Institute research platform, which is supported by the UNSW servers. The school counsellor receives real-time notifications for students who require follow-up via a secure, purpose-built Web-based portal. The school counsellor can log into the portal to identify the students requiring follow-up and utilize their usual duty of care and referral pathways to treat students. Using the portal functions, school counsellors can notify the research team that follow-ups have been completed. There is also the option for counsellors to provide case notes, detailing treatment action and any adverse events. School counsellors are also able to view these participants’ use of the Smooth Sailing program, including module completion. School counsellors receive an automated email reminder to check the portal after every assessment point. Researchers also receive email notifications for any student who triggers a notification. No identifiable information is contained in these emails.

### Control

This is a school-as-usual condition. As such, students can access the school counsellor as needed and are permitted to participate in any mental health education or activities initiated by the school or external mental health professionals. Schools allocated to the control condition are placed on a wait list to receive the Smooth Sailing service after the trial data collection period has been completed.

### Procedure

At baseline, researchers from the Black Dog Institute visit the school to conduct the study during class time. After providing a verbal explanation of the study and confirming consent, students are asked to use any internet device to visit the study website. Instructions for registering and completion of study questionnaires are provided to students on paper. The researchers remain with students to provide assistance and answer any questions. At 6 weeks and 12 weeks, researchers revisit the schools and repeat this process. Students who are unable to access the website at 6 weeks or 12 weeks because of technical issues are given a paper copy of the questionnaires to complete. If these students are the intervention arm, researchers use a paper scoring method to calculate participants’ step at these timepoints and inform the school counsellor verbally if the student requires follow-up. The research team then enters these responses into the system on return to the office resulting in the immediate update of the Web-based portal. Researchers meet with the school counsellor after each visit to review the students who require follow-up and to ensure that counsellors feel adequately supported to conduct these. The researchers contact the school counsellors 2 working days after each visit to review student follow-ups and monitor any adverse events.

**Table 4:** Stepping decisions at 6 weeks and 12 weeks.

Current step (symptom severity: score range)	Previous step				
	0	1	2	3	4
0 (Minimal: 0-4)	0	1	2	3	4
1 (Mild: 5-9)	1	1	2	3	4
2 (Moderate: 10-14)	2	2	3	3	4
3 (Moderately severe: 15-19)	3	3	3	4	4
4 (Severe: 20+)	4	4	4	4	4

### Sample Size

The target sample size for this trial is 1600. This calculation is based on detecting an effect size of 0.20, which is similar to that obtained in previous school-based depression intervention research [9]. The statistical power level is set at 0.8, alpha=.05 (2-tailed), whereas a correlation of 0.5 is assumed between the scores at baseline and endpoint. A design effect is calculated assuming an intraclass correlation of 0.02 and an average school sample of 300 students to allow for possible clustering effects. This estimate was derived from previous Australian school–based studies [9]. The estimated sample size also accommodates for a 20% attrition rate based on previous school-based trials [43]. To achieve the desired sample size and ensure representation from a range of different types of schools, the target is set at 16 schools with approximately 100 students participating per school.

### Outcome Measures

The administration schedule for the outcome measures (Multimedia Appendix 1) is outlined in Table 5.

#### Demographics

At baseline, students provide their name, email, mobile phone number (optional), gender, and date of birth. Students are asked to report their current employment status (answered part-time, casual, or nil) and whether they identify as Aboriginal and or Torres Strait Islander (answered yes, no, I’d rather not say) or Lesbian, Gay, Bisexual, Trans or Intersex (answered yes, no, I’d rather not say).

#### Mental Health History

At baseline, participants are asked to report whether they have ever known someone with a mental illness (answered yes, no, I’m not sure); cared for someone with a mental illness (answered yes, no, I’m not sure); or if they have previously had a session with the school counsellor at their school (answered yes, no, I’d rather not say). Participants are also asked if they have ever used the internet to find information about a mental health problem (answered yes, no, I’m not sure) and whether they think doing a Web-based program will help their mental health (answered yes, no, I’d rather not say). At baseline, 6 weeks, and 12 weeks, participants are also asked if they have been diagnosed with a mental health problem or mental illness, if they have received any treatment for a mental health problem or mental illness, or if they have taken any prescribed medication (eg, antidepressants) for a mental health problem or mental illness (answered yes, no, I’d rather not say).

### Primary Outcome Measure

#### General Help-Seeking Questionnaire

The General Help-Seeking Questionnaire (GHSQ) is used to measure help-seeking intentions. Participants are asked to rate how likely they are to seek help from 13 different sources (friend, partner, parent, other family member, teacher, other adult, school counsellor, doctor/general practitioner, mental health professional, telephone helpline, mental health website, a Web-based mental health program, and other internet activity) if faced with a mental health problem, with each item answered on a 5-point scale ranging from extremely unlikely to extremely likely [27]. Participants can also indicate if they would not seek help from anyone at all or if they would seek help from someone not listed. The GHSQ has demonstrated satisfactory psychometric properties for measuring the likelihood of seeking help among adolescents [27]. In this study, a *total help-seeking intentions score* is calculated for each participant consisting of their responses to the 13 sources, with total possible scores ranging from 14 to 70.

**Table 5:** Outcome measures administration schedule.

Assessment		Construct	Baseline	6 weeks	12 weeks
**Primary outcome**					
	General Help-Seeking Questionnaire	Intentions to seek help	Yes	No	Yes
**Secondary outcomes**					
	Actual Help-Seeking Questionnaire	Actual help-seeking behavior	Yes	Yes	Yes
	Generalized Anxiety Disorder Questionnaire	Generalized anxiety	Yes	Yes	Yes
	Center for Epidemiologic Studies Depression Scale—Child Version	Depressive symptoms	Yes	Yes	Yes
	Functioning	Functioning	Yes	Yes	Yes
	Distress Questionnaire-5	Psychological distress	Yes	Yes	Yes
	Barriers to Adolescents Seeking Help-Brief	Barriers to seeking help	Yes	No	Yes
	Mental Health Literacy and Stigma Scale	Mental health literacy and stigma	Yes	No	Yes
**Tertiary outcome**					
	Satisfaction questionnaire^a^	Service adherence and satisfaction	No	No	Yes

^a^Delivered to Intervention schools only.

### Secondary Outcome Measures

#### Actual Help-Seeking Questionnaire

The Actual Help-Seeking Questionnaire is used to assess help-seeking behavior [44]. Using the same sources presented in the GHSQ, participants are asked to report whether they have sought help for a mental health problem from these sources in the past 6 weeks (answered yes or no). Participants can also indicate if they have sought help from someone not listed. An additional question asks participants whether they have needed support for their mental health but did not seek help. The use of this questionnaire in youth has been well supported [30]. In this study, participants’ responses at baseline and 12 weeks posttest are categorized into a new variable named *help-seeking from adults*. Participants who have sought help from any adult source are categorized as yes, with those who have not categorized as no. This variable is then used as a secondary outcome for analyses, alongside the number of participants who report not seeking help at all despite having a need for mental health support.

#### Generalized Anxiety Disorder Questionnaire

Generalized anxiety is measured using the GAD-7 [22], which is a brief measure of anxiety consisting of 7 items that are rated on a scale from not at all (0) to nearly every day (4). Total scores can be classified as nil to mild (5-9), moderate (10-14), or moderately severe to severe (>15). A score of 10 and above is recommended as a reasonable cutoff point for identifying cases of clinical anxiety [22]. The GAD-7 has been found to have good test-retest reliability and strong criterion validity [22].

#### Center for Epidemiologic Studies Depression Scale—Child Version

The Center for Epidemiologic Studies Depression Scale (CES-D) was originally developed for measuring depression in an adult population [45] and was later modified into the child version, the Center for Epidemiologic Studies Depression Scale – Child Version (CES-DC) [46]. It is comprised 20 items, rated from not at all (0) to a lot (3), with 4 items reverse-scored. The total possible scores range from 0 to 60, with higher scores indicative of increased levels of depression. The scale has been found to have good psychometric properties among adolescents [47]. The most commonly used cutoff score for the CES-D is 16 and above, which indicates the individual should be further screened for risk of depression [48].

#### Distress Questionnaire-5

The Distress Questionnaire-5 (DQ5) is a brief, valid screener used for identifying those at risk of psychological distress in the general population [49]. The DQ5 consists of 5 items rated from never (1) to always (5). Total scores range from 5 to 25 and higher scores indicate greater psychological distress. It has been suggested that the DQ5 may be superior to the K6 and K10 in identifying increased risk for common mental disorders, with a screening cutoff point of 11 indicating the likely presence of a mental health condition [49].

#### Functioning

Taken at baseline and posttest, this is a 1-item question in which students are asked to rate how difficult their symptoms of anxiety and depression have made their daily life and relationships. Participants answer using a 4-point Likert scale ranging from not at all difficult (0) to very difficult (4).

#### Barriers to Adolescents Seeking Help

The Barriers to Adolescents Seeking Help-Brief [50] is a shortened (11 items) version of the original questionnaire, measuring barriers to help-seeking behavior among adolescents. Participants rate their level of agreement with a list of statements about help-seeking from strongly disagree (1) to strongly agree (6). Total scores range from 11 to 66, with higher mean scores reflecting greater resistance to seeking professional help. The scale has been found to have good test-retest reliability and validity in a population of high school students [50].

#### Mental Health Literacy and Stigma Scale

This scale measures a person’s confidence about help-seeking for mental health and stigmatizing attitudes toward mental illness. It has demonstrated good internal and test-retest reliability and validity [51]. A shortened version of the questionnaire, consisting of 4 items assessing confidence seeking help and 9 items assessing stigmatizing attitudes of mental illness, has been selected for use in this study. Item responses are rated on a 5-point scale from strongly disagree (1) to strongly agree (5) with 9 items reverse-scored. Total scores for this scale range from 13 to 65, with higher scores indicating greater confidence in seeking help for mental health problems and lower levels of stigma.

### Tertiary Outcome Measure

#### Service Adherence and Satisfaction

Service adherence is measured by the number of modules accessed and the number of check-ins completed by the intervention participants. A total of 3 questionnaires assess service satisfaction. The first questionnaire assesses 3 main domains: enjoyment and ease of use, usefulness, and degree of comfort with service requirements. This questionnaire requires participants to rate whether they agree or disagree with 11 statements about the service, for example, *I enjoyed using Smooth Sailing*. The second questionnaire asks participants to report on 18 service use barriers across 3 key domains: technical, personal, or intervention-specific (answered yes or no), for example, *My internet connection didn’t work*. The third questionnaire examines participants’ use of the websites and CBT programs suggested by the service (answered yes, no, or I can’t remember). Participants are also asked to report if they have been contacted by the school counsellor during the study period and if they felt comfortable when this occurred. Participants are also asked to provide an overall rating of helpfulness of the service (answered on a Likert scale of 1 to 5 from extremely unhelpful to extremely helpful) and their suggestions for improvements or any other comments (2 free response options).

### Assessment of Safety

#### Data Monitoring

A data safety monitoring board is not utilized because the study targets a nonclinical population, blinding is not used, and the service provides a real-time monitoring and notification system of participants’ thoughts of death and self-harm. An Outcomes Advisory Group has been established to provide specific monitoring, governance, and reporting of adverse events and trial safety. This consists of the CI, chief scientist of the Black Dog Institute, a medical expert in child and adolescent psychiatry, and a clinical psychologist. This team meets monthly, and on an as-needed basis, to monitor the safety of the trial. During baseline, 6-week, and 12-week assessments, the research team notifies the Outcomes Advisory Group by email every fortnight of the total number of students participating and the notifications received for each school. This group is also responsible for responding to any complaints.

#### Harms

Throughout the study period, the duty of care for all students remains with their participating school. A trained mental health researcher from the Black Dog Institute is present at all school visits, alongside a school staff member. The school counsellor and a private breakout space is made available for any students who become distressed during the assessments. In the control condition, all students are provided with an information sheet of help-seeking resources and services at baseline, 6-week, and 12-week assessment. In the intervention condition, the Smooth Sailing service includes a question assessing thoughts of dying or harming oneself. This is part of the PHQ-9 screening measure for depression and aims to provide an objective measure of symptom severity if completed truthfully. However, further assessment from a trained mental health professional is required for the extent of an individual’s suicide risk to be fully determined. If a student reports the presence of these thoughts during the assessments, information about suicide support services is immediately provided by the service through a pop-up. The school counsellors are also notified using the Web-based portal and email system. The school counsellors are also notified when any participant in the intervention condition reports severe depression or anxiety symptoms, or there is risk of significant harm as identified by the school. The counsellors are notified to conduct the follow-up assessments to determine participants’ risk, using normal school protocols. A list of local services and resources is provided to the school counsellors to assist them in following up with students. The child and adolescent psychiatrist in the Outcomes Advisory Group is also available to provide advice and assistance to the school counsellors throughout the study period. School counsellors are asked to report on follow-ups to the research team via phone call or email with a record-keeping option on the school counsellor Web portal. The research team also inquire about adverse events at each of the school visits and report on these in their fortnightly reports to the Outcomes Advisory Group. At the final study visit, all students are provided with a list of mental health resources and services information.

### Data Handling, Storage, and Access

The study is hosted on the servers of the Black Dog Institute Research Platform, Faculty of Medicine, UNSW. These servers are encrypted with data backups occurring daily. This platform and its associated data are only accessible to authorized and approved personnel. When registering, participants create password-protected accounts and the platform allocates a unique identification number. For analyses, data are deidentified by removing names, email address, and mobile phone numbers. Students who actively withdraw will have their data removed and a withdrawal confirmation email will be sent from the research team. The platform retains data from the students who are lost to follow-up. All data will be stored securely for a minimum of 15 years.

### Auditing

Researchers follow step-by-step guidelines for each study visit to ensure consistency across different trial sites and research team members. A decision-making guide for trial conduct is provided to all research staff. School packages are mailed to schools ahead of time, which included trial documents, such as an explanatory paragraph for the school newsletter, an agenda for the baseline visit, and a list of local support services, to facilitate familiarity with the consent process and study procedure. Instructional documents for intervention school counsellors are provided to ensure adherence with the study protocol and to guide their use of the Web-based portal. All research staff debrief with the CI or research manager after each school visit. Fortnightly research team meetings are held to discuss and audit the conduct of the trial. The research team also have frequent contact with the school counsellors to assess any adverse events.

### Analysis

Data are collected using the Black Dog Institute electronic health platform. Data will be downloaded into Microsoft Excel and exported to SPSS Version 22.0 (SPSS Inc) for analysis. Primary analyses will be undertaken on an intention to treat basis, including all participants randomized, regardless of treatment received. Effectiveness of Smooth Sailing will be established by a change in the GHSQ scores between baseline and 12 weeks using a mixed-effects model repeated-measures analysis. The school will be included in analyses as a random effect to evaluate and accommodate clustering effects. In analyses of scaled secondary variables, methods comparable with those of the primary analysis will be used. An analysis of covariance will be conducted for the secondary outcomes of anxiety, depression, distress, functioning, mental health literacy, and barriers to help-seeking, controlling for baseline scores. To determine the effect of the intervention on help-seeking, 2 binary logistic regression analyses will be used. In model 1, the dependent variable will be help-seeking from adults at 12 weeks. The baseline result will be entered at step 1 in the model, with the study arm entered at step 2. In model 2, the dependent variable will be not seeking help at all at 12 weeks despite a need. The baseline result will be entered at step 1, with the study arm entered at step 2.

### Dissemination Policy

A summary of the results will be emailed to all participants who request this on their consent form. The results summary will also be published on the Black Dog Institute website. School reports, consisting of the mean aggregate scores for the measures, will be prepared and shared with the school at completion of the data collection period. The primary and secondary outcomes analysis will be prepared for academic publication and presentations. A final report will also be submitted to the funding body. In all documents, participants will not be individually identifiable with data presented at the aggregate level.

## Results

The results for this trial are currently under analysis. Ethics approval was provided by UNSW HREC on November 24, 2017, and by the NSW Department of Education SERAP on January 10, 2018. An Expression of Interest for the trial was advertised through the NSW School-Link Initiative (School-Link) newsletter and email. We were subsequently contacted by a school representative (eg, School Counsellor, Head of Well-Being) from 88 different schools. Throughout January and February 2018, 22 secondary schools across NSW agreed to participate in the trial. These schools were randomized into 2 conditions, with 10 schools assigned to receive the intervention and 12 assigned to the control condition. The first baseline assessment occurred on February 22, 2018, with the 12-week endpoint assessments completed on Friday, June 29, 2018. Control schools are currently receiving the service, due for completion by June 30, 2019.

## Discussion

This study protocol outlines the Smooth Sailing trial, which aims to investigate the effectiveness of a Web-based mental health service for secondary school students. Using a randomized controlled design, this study evaluates the efficacy of the Smooth Sailing service for improving a range of mental health outcomes among students compared with school-as-usual. The service was designed to increase adolescents’ help-seeking intentions for mental health problems, as well as to improve help-seeking behaviors and reduce symptoms of anxiety, depression, and distress. This is critically important to this age group as mental illness first emerges during adolescence and help-seeking from professionals is low [2,3]. Most notably, this study represents the first attempt at delivering a school-based mental health service that incorporates the principles of stepped care with Web-based screening and intervention alongside direct face-to-face follow-up.

During the development of the service, school counsellors, parents, and general practitioners (GPs) outlined various concerns related to the accuracy, effectiveness, and suitability of the service for delivery in the school setting [18-20]. Some parents questioned the effectiveness of a Web-based service for determining the level of care their child required [20] and whether the service would increase stigma among students. Both parents and school counsellors were concerned that students may not openly disclose their symptoms to a Web-based service [18]. Parents, counsellors, and GPs [19] were also concerned about teens’ adherence and engagement with self-directed Web-based CBT programs. This study will be able to determine these outcomes. This trial will also evaluate the benefits of using a Web-based service to initiate school counsellor referrals for symptomatic students compared with traditional referral processes, and the capacity of school counsellors to manage these. This study will also be able to examine the effectiveness of school counselling services in Australia, an area in which effectiveness data are lacking.

The results of this trial will confirm whether an integrated service model that combines screening, triage, intervention, and monitoring is acceptable and effective in the school environment on a large scale. A multicenter trial of a suicide prevention program in Europe revealed that screening alone was not effective for improving outcomes [52]. The authors argued that concurrent activities are needed, including mental health literacy and broader awareness among the student year group. Furthermore, adolescents were more likely to engage in programs and services that acknowledge their autonomy, rather than being adult or teacher driven. These findings have important implications for this trial, suggesting that the Smooth Sailing service is likely to influence students’ mental health outcomes. Overall, the trial will determine whether universal screening with prompted access to Web-based psychoeducation, self-directed CBT and school counselling services will increase help-seeking among secondary school students.

Although the co-design process and pilot study indicated that Smooth Sailing was acceptable and feasible to deliver, practical considerations for the larger trial were outlined. Students revealed that they would disapprove of this type of service being made compulsory at school and instead felt engagement and interest in the service could be increased if an opt-out parental consent process was implemented. This was supported by school staff as they felt it respected students’ increasing autonomy and addressed privacy concerns, especially among students who were more reluctant to participate in mental health initiatives or were experiencing mental health problems. To increase student engagement in the service content, students and staff requested the use of multimodal reminders. For example, school staff could be encouraged to remind students to use Smooth Sailing for a certain amount of time per week, alongside SMS text messaging and email reminders for students at fortnightly intervals. It was also suggested that the Web-based psychoeducation content be modified and reduced. School counsellors also felt that a Web-based portal would increase their efficiency by improving their ability to keep track of follow-ups and better understand the mental health care needs of those students who trigger a notification. All of these changes have been made and instigated in the current protocol.

Certain limitations may affect the findings in this study. Given that the intervention condition involves being followed up by the school counsellor, it may be that students from the intervention schools are less likely to consent than students from the control schools. Furthermore, the increased contact with students in the intervention arm through the service check-ins may impact retention. If found to be effective, the researchers will need to carefully consider the range of implementation barriers that challenge school-based mental health initiatives, including maintaining service fidelity and access beyond trial completion [12]. Major strengths of the study include the RCT design, the novelty of the intervention, and the use of stepped care in the school context. This study will be able to determine the usefulness and effectiveness of a stepped care model, confirming whether a 5-step model is justified. The trial findings will provide vital information about whether this new and innovative model of care for youth mental health is effective for when delivered in the school setting.

## Appendix

Multimedia Appendix 1 Outcome measures schedule.

## Abbreviations

CBT: Cognitive Behavioral Therapy
CI: Chief Investigator
DQ5: Distress Questionnaire-5
GAD-7: Generalized Anxiety Disorder Scale-7
GHSQ: General Help-Seeking Questionnaire
GPs: General Practitioners
HREC: Human Research Ethics Committee
NSW: New South Wales
PHQ-9: Patient Health Questionnaire-9
PICF: Participant Information and Consent Form
RCT: Randomized Controlled Trial
SCS: Sydney Catholic Schools
SERAP: State Education Research Applications Process
SMS: Short Message Service
UNSW: University of New South Wales
