# Supplementary material for: Evaluating a Web-Based Mental Health Service for Secondary School Students in Australia: Protocol for a Cluster Randomized Controlled Trial
Source: JMIR Res Protoc. 2019 May 17;8(5):e12892. doi: 10.2196/12892 (PMC6543801; doi:10.2196/12892)
Supplement: Multimedia Appendix 1 [file resprot_v8i5e12892_app1.pdf]

Outcome Measures

Personal Information Questionnaire

We'd love it if you could tell us a little about yourself...			
Do you identify as being Lesbian, Gay, Bisexual, Trans, or Intersex?	☐ Yes	☐ No	☐ I'd rather not say
Do you identify as being Aboriginal or Torres Strait Islander?	☐ Yes	☐ No	☐ I'd rather not say
Are you currently working?	☐Yes - Part time job/s	☐Yes - Part casual job/s	☐ No, not currently working
Have you ever known anyone with a mental health problem or mental illness?	☐ Yes	☐ No	☐ I'm not sure
If yes, who? Circle all that apply	Friend Parent Sibling Girlfriend/Boyfriend Other		
Have you ever helped look after someone with a mental illness or mental health problem?	☐ Yes	☐ No	☐ I'm not sure
If yes, who? Circle all that apply	Friend Parent Sibling Girlfriend/ Boyfriend Other		
Have you ever had a mental health problem or been diagnosed with a mental illness?*	☐ Yes	☐ No	☐ I'd rather not say
Are you currently receiving any treatment for a mental health problem?*	☐ Yes	☐ No	☐ I'd rather not say
Are you currently taking any medication (e.g. anti-depressants) for a mental health problem?*	☐ Yes	☐ No	☐ I'd rather not say
Have you ever had a private session with the school counsellor at your school?	☐ Yes	☐ No	☐ I'd rather not say
Have you ever used the Internet to find information or get some help for a mental health problem?	☐ Yes	☐ No	☐ I'd rather not say
If yes, did you find it helpful?	☐ Yes	☐ No	☐ I can't remember
Do you think doing an online program will help your mental health?	☐ Yes	☐ No	☐ I'm not sure

**asked again at 6 and 12-week follow-up*

Generalised Anxiety Disorder Questionnaire (GAD-7)

**Over the last two weeks, how often have you been bothered by the following problems?**	**Not at all**	**Several days**	**Over half the days**	**Nearly every day**
Feeling nervous, anxious, or on edge	0	1	2	3
Not being able to stop or control worrying	0	1	2	3
Worrying too much about different things	0	1	2	3
Trouble relaxing	0	1	2	3
Being so restless that it is hard to sit still	0	1	2	3
Becoming easily annoyed or irritable	0	1	2	3
Feeling afraid as if something awful might happen	0	1	2	3

Functioning

	**Not at all**	**Somewhat difficult**	**Very difficult**	**Extremely difficult**
**How difficult have these problems made it for you to do your school work, take care of things at home, or get along with your mates and family?**	☐	☐	☐	☐

Centre for Epidemiologic Studies Depression Scale – Child version (CES-DC)

**Think back over the past week. Tell us how often the following happened…**	**Not at all**	**Several days**	**More than half the days**	**Nearly every day**
I was bothered by things that usually don't bother me	☐	☐	☐	☐
I did not feel like eating, I wasn't very hungry	☐	☐	☐	☐
I wasn't able to feel happy, even when my family or friends tried to help me feel better	☐	☐	☐	☐
I felt like I was just as good as other kids	☐	☐	☐	☐
I felt like I couldn't pay attention to what I was doing	☐	☐	☐	☐
I felt down and unhappy	☐	☐	☐	☐
I felt like I was too tired to do things	☐	☐	☐	☐
I felt like something good was going to happen	☐	☐	☐	☐
I felt like things I did before didn't work out right	☐	☐	☐	☐
I felt scared	☐	☐	☐	☐
I didn't sleep as well as I usually sleep	☐	☐	☐	☐
I was happy	☐	☐	☐	☐

I was more quiet than usual	☐	☐	☐	☐
I felt lonely, like I didn't have any friends	☐	☐	☐	☐
I felt like kids I know were not friendly or that they didn't want to be with me	☐	☐	☐	☐
I had a good time	☐	☐	☐	☐
I felt like crying	☐	☐	☐	☐
I felt sad	☐	☐	☐	☐
I felt people didn't like me	☐	☐	☐	☐
It was hard to get started doing things	☐	☐	☐	☐

Distress Questionnaire-5 (DQ5)

**Think back over <u>the last 30 days</u>. Tell us how often the following happened…**	**Never**	**Rarely**	**Sometimes**	**Often**	**Always**
My worries overwhelmed me	☐	☐	☐	☐	☐
I felt hopeless	☐	☐	☐	☐	☐
I found social settings upsetting	☐	☐	☐	☐	☐
I had trouble staying focused on tasks	☐	☐	☐	☐	☐
Anxiety or fear interfered with my ability to do the things I needed to do at school or at home	☐	☐	☐	☐	☐

General Help-Seeking Questionnaire (GHSQ)

**If you were having a tough time with your mental health, how likely is it that you would seek help from...**	**Extremely unlikely**	**Unlikely**	**Neither**	**Likely**	**Extremely likely**
A friend	☐	☐	☐	☐	☐
A boyfriend or girlfriend	☐	☐	☐	☐	☐
Parents	☐	☐	☐	☐	☐
Other relative or family member (e.g. brothers/sisters, aunts/uncles, grandparents)	☐	☐	☐	☐	☐
A teacher	☐	☐	☐	☐	☐
Other adult (e.g. sports coach, a mate's parent, a work mate)	☐	☐	☐	☐	☐
A school counsellor	☐	☐	☐	☐	☐
General Practitioner/Local Doctor	☐	☐	☐	☐	☐

A mental health professional (e.g. psychologist or psychiatrist)	☐	☐	☐	☐	☐
A telephone helpline (e.g. Kids Helpline, Lifeline)	☐	☐	☐	☐	☐
A website on mental health (e.g. ReachOut)	☐	☐	☐	☐	☐
Other internet activity (e.g. online discussion forums, social media)	☐	☐	☐	☐	☐
Someone not listed above_______________	☐	☐	☐	☐	☐
I would not seek help from anyone	☐	☐	☐	☐	☐

Actual Help-Seeking Questionnaire (AHSQ)

**In the past three months, have you turned to any of the following for help with a mental health issue...**	**Yes**	**No**
Friend	☐	☐
Boyfriend or Girlfriend	☐	☐
Parent	☐	☐
Other relative or family member (e.g. brothers/sisters, aunts/uncles, grandparents)	☐	☐
Other adult (e.g. sports coach, a mate's parent, a work mate)	☐	☐
Teacher	☐	☐
School Counsellor	☐	☐
General Practitioner/Local Doctor	☐	☐
A mental health professional (e.g. psychologist or psychiatrist)	☐	☐
Telephone Helpline (e.g. Kids Helpline, Lifeline)	☐	☐
A Mental Health website (e.g. ReachOut)	☐	☐
An online mental health program (e.g. MoodGym, Smooth Sailing, Brave Online)	☐	☐
Other internet activity (e.g. online discussion forums, social media)	☐	☐
Someone else not listed above: _________________________________	☐	☐
I needed support but did not seek help from anyone	☐	☐

Barriers to Adolescents Seeking Help (BASH-Brief)

**How much do you agree with the following statements?**	**Strongly Disagree**	**2**	**3**	**4**	**5**	**Strongly Agree**
I would solve my problems myself	☐	☐	☐	☐	☐	☐

I think I should work out my own problems	☐	☐	☐	☐	☐	☐
I'd be too embarrassed to talk to a counsellor	☐	☐	☐	☐	☐	☐
Adults can't understand adolescent problems	☐	☐	☐	☐	☐	☐
Even if I wanted to, I wouldn't have time to see a counsellor	☐	☐	☐	☐	☐	☐
A counsellor might make me do what I don't want to do	☐	☐	☐	☐	☐	☐
I wouldn't want my family to know I was seeing a counsellor	☐	☐	☐	☐	☐	☐
I couldn't afford to see a counsellor	☐	☐	☐	☐	☐	☐
Nothing will change the problems I have	☐	☐	☐	☐	☐	☐
If I go to counselling, I might find out I'm crazy	☐	☐	☐	☐	☐	☐
If I went for help, the counsellor would not keep my secret	☐	☐	☐	☐	☐	☐

Mental Health Literacy and Stigma Scale (MHLS)

**How much do you agree with the following?**	**Strongly Disagree**	**Disagree**	**Neither**	**Agree**	**Strongly Agree**
I am confident that I know where to seek information about mental health	☐	☐	☐	☐	☐
I am confident using the computer or telephone to seek information about mental health	☐	☐	☐	☐	☐
I am confident attending face to face appointments to seek information about mental health (e.g., seeing the GP)	☐	☐	☐	☐	☐
I am confident I have access to resources (e.g., GP, internet, friends) that I can use to seek information about mental health	☐	☐	☐	☐	☐
People with a mental illness could snap out of it if they wanted	☐	☐	☐	☐	☐
A mental illness is a sign of personal weakness	☐	☐	☐	☐	☐
A mental illness is not a real medical illness	☐	☐	☐	☐	☐

People with a mental illness are dangerous	☐	☐	☐	☐	☐
It is best to avoid people with a mental illness so that you don't develop this problem	☐	☐	☐	☐	☐
If I had a mental illness I would not tell anyone	☐	☐	☐	☐	☐
Seeing a mental health professional means you are not strong enough to manage your own difficulties	☐	☐	☐	☐	☐
If I had a mental illness, I would not seek help from a mental health professional	☐	☐	☐	☐	☐
I don't think treatment for a mental illness, provided by a mental health professional, would work	☐	☐	☐	☐	☐

Service Adherence and Satisfaction Questionnaire for Intervention participants

Part 1:

We'd like to see what you thought about Smooth Sailing. This will help us improve the program.

**Please tell us whether you agree or disagree with the following statements**	**Agree**	**Disagree**
I enjoyed using Smooth Sailing	☐	☐
I thought Smooth Sailing was interesting	☐	☐
I felt comfortable providing my mobile phone number	☐	☐
I felt comfortable providing my email address	☐	☐
I found Smooth Sailing easy to use	☐	☐
Smooth Sailing was easy to understand	☐	☐
Smooth Sailing helped me to feel in control of my feelings	☐	☐
The skills I learned from Smooth Sailing helped me a lot in everyday life	☐	☐
I understood that I might be followed up by the school counsellor and was okay with this	☐	☐
I would use Smooth Sailing again in the future	☐	☐
I would tell a friend to use Smooth Sailing if I thought they needed to	☐	☐

Part 2:

We'd like to know if you had any trouble using Smooth Sailing.

**During the study, did any of these things happen to you?**	**Yes**	**No**
My internet connection didn't work	☐	☐

I forgot how to access Smooth Sailing	☐	☐
I didn't have time to use Smooth Sailing	☐	☐
I couldn't be bothered to use Smooth Sailing	☐	☐
I felt too worried or too down to use Smooth Sailing	☐	☐
I didn't want my school counsellor to know how I was feeling	☐	☐
I didn't have a phone or computer to use	☐	☐
I just forgot it was there	☐	☐
I felt Smooth Sailing wasn't what I needed	☐	☐
I was worried about the privacy of my data	☐	☐
I didn't trust Smooth Sailing	☐	☐
I thought that the content took too long to read	☐	☐
I thought the text was too small and too hard to read on my phone	☐	☐
I thought it took too long to load	☐	☐
I thought the check-ins took too long to complete	☐	☐
I thought Smooth Sailing used up too much of my phone data	☐	☐
I had trouble logging into the website	☐	☐
Smooth Sailing made me feel worse	☐	☐

Part 3:

Smooth Sailing suggests that you try a range of different websites and online programs. We'd like to know if you used any of these.

**In the last 12 weeks, did you...**	**Yes**	**No**	**I can't remember**
Use a website called MoodGym?	☐	☐	☐
If No, why didn't you?			
Use a website called Brave Online?	☐	☐	☐
If No, why didn't you?			
Visit any of the other websites that Smooth Sailing suggested?	☐	☐	☐
If No, why didn't you?			
**In the last 12 weeks…**	**Yes**	**No**	**I can't remember**
Were you contacted by your school counsellor in the last 12 weeks?	☐	☐	☐
If Yes, did you feel uncomfortable when this happened?	☐	☐	☐

	**Extremely unhelpful**	**Unhelpful**	**Neither**	**Helpful**	**Extremely helpful**
Overall, how helpful was Smooth Sailing?	☐	☐	☐	☐	☐
If Helpful or Extremely helpful, in what ways did Smooth Sailing help you?					

What could we do to make Smooth Sailing better?

Is there anything else you'd like to tell us about Smooth Sailing? (Leave blank if no!)

